# Identification and Predicting Short-Term Prognosis of Early Cardiorenal Syndrome Type 1: KDIGO Is Superior to RIFLE or AKIN

**DOI:** 10.1371/journal.pone.0114369

**Published:** 2014-12-26

**Authors:** Zhilian Li, Lu Cai, Xinling Liang, Zhiming Du, Yuanhan Chen, Shengli An, Ning Tan, Lixia Xu, Ruizhao Li, Liwen Li, Wei Shi

**Affiliations:** 1 Department of Nephrology, Guangdong General Hospital, Guangdong Academy of Medical Sciences, Guangzhou, China; 2 Department of Nephrology, Huzhou Central Hospital, Huzhou, China; 3 Southern Medical University, Guangzhou, China; 4 Department of Cardiology, the First Affiliated Hospital, Sun Yat-sen University, Guangzhou, China; 5 Department of Bio-Statistics, Southern Medical University, Guangzhou, China; 6 Department of Cardiology, Guangdong Cardiovascular Institute, Guangdong General Hospital, Guangzhou, China; University of São Paulo State - Botucatu School of Medicine - UNESP, Brazil

## Abstract

**Objective:**

Acute kidney injury (AKI) in patients hospitalized for acute heart failure (AHF) is usually type 1 of the cardiorenal syndrome (CRS) and has been associated with increased morbidity and mortality. Early recognition of AKI is critical. This study was to determine if the new KDIGO criteria (Kidney Disease: Improving Global Outcomes) for identification and short-term prognosis of early CRS type 1 was superior to the previous RIFLE and AKIN criteria.

**Methods:**

The association between AKI diagnosed by KDIGO but not by RIFLE or AKIN and in-hospital mortality was retrospectively evaluated in 1005 Chinese adult patients with AHF between July 2008 and May 2012. AKI was defined as RIFLE, AKIN and KDIGO criteria, respectively. Cox regression was used for multivariate analysis of in-hospital mortality.

**Results:**

Within 7 days on admission, the incidence of CRS type 1 was 38.9% by KDIGO criteria, 34.7% by AKIN, and 32.1% by RIFLE. A total of 110 (10.9%) cases were additional diagnosed by KDIGO criteria but not by RIFLE or AKIN. 89.1% of them were in Stage 1 (AKIN) or Stage Risk (RIFLE). They accounted for 18.4% (25 cases) of the overall death. After adjustment, this proportion remained an independent risk factor for in-hospital mortality [odds ratios (OR)3.24, 95% confidence interval(95%CI) 1.97–5.35]. Kaplan-Meier curve showed AKI patients by RIFLE, AKIN, KDIGO and [K(+)R(−)+K(+)A(−)] had lower hospital survival than non-AKI patients (Log Rank P<0.001).

**Conclusion:**

KDIGO criteria identified significantly more CRS type 1 episodes than RIFLE or AKIN. AKI missed diagnosed by RIFLE or AKIN criteria was an independent risk factor for in-hospital mortality, indicating the new KDIGO criteria was superior to RIFLE and AKIN in predicting short-term outcomes in early CRS type 1.

## Introduction

Acute kidney injury (AKI) is common and one of the most powerful determinants of outcome in acute heart failure (AHF) [1]–[3]. According to a recently published classification, AKI after hospitalization for AHF is usually characteristic of the acute (Type 1) cardiorenal syndrome (CRS) [4]–[6]. Early recognition of AKI is critical in AHF [7]. Indeed, worsening renal function after hospitalization for AHF is frequently observed and has been a predictor of longer hospital stay and increased mortality [1]–[3].

The definition of AKI was recently revised. The first consensus classification of AKI, known as the RIFLE criteria, was defined based on a ≥50% increase in serum creatinine (SCr) level occurring over 1–7 days or the presence of oliguria for more than 6 hours [8]. The RIFLE criteria subsequently were modified by the AKI Network (AKIN) in 2007, by the addition of an absolute increase in SCr level of 0.3 mg/dL and reduced the timeframe for the increase in SCr level to 48 hours [9]. The diagnosis of AKI may be missed when using one or the other classification schemes [10]. Thus combining the two criteria ensures that the diagnosis is capture. The most recent consensus definition proposed by the Kidney Disease Improving Global Outcomes (KDIGO) Work Group in 2012 [11], harmonizing RIFLE and AKIN definitions, contains those individuals diagnosed as AKI but not by RIFLE or AKIN. However, the new KDIGO criterion was not yet widely validated. More importantly, it remains unclear whether the proportion of AKI diagnosed by KDIGO criteria but missed by RIFLE or AKIN is associated with an increased risk of death during hospitalization. This study was to evaluate the incidence of unidentified AKI by RIFLE or AKIN criteria and their prognostic impact in AHF patients. We hypothesize that KDIGO is superior to RIFLE and AKIN criteria in predicting in-hospital mortality in the setting of early CRS type 1 (within 7 days on admission).

## Patients and Methods

### Study Cohort

This retrospective cohort study was conducted at Guangdong General Hospital and the First Affiliated Hospital of Sun Yat-sen University in Guangzhou, China. We collected 1,498 adult patients (aged ≥18 years) hospitalized with acute heart failure (AHF) between July 2008 and July 2012. AHF was defined as either now-onset HF or decompensation of chronic HF with symptoms sufficient to warrant hospitalization. The diagnosis of AHF was based on European Society of Cardiology Criteria [12]. All patients had a New York Heart Association (NYHA) functional class of either Class III or IV. Only the first hospital admission was considered if a patient had more than one hospitalization for AHF during the study period. Patients were excluded if they met the following exclusion criteria: lack of SCr measurement during the first 7 days of hospitalization, early death within 48 h after admission, hospital stay <48 h, end-stage renal disease with dialysis, admission SCr level ≥3.5 mg/dl, malignant tumor, cardiac surgery- or contrast medium-associated AKI.

The Ethics Research Committee at Guangdong General Hospital and the First Affiliated Hospital of Sun Yat-sen University approved the study and agreed that informed consent was not necessary because of the purely observational nature of this study. Information of all patients was anonymized and de-identified prior to analysis.

### Definition and Staging of AKI

We defined and staged AKI according to SCr-based criteria per the RIFLE, AKIN and KDIGO criteria within the first 7 days of hospitalization (Table 1). Here the RIFLE classification only included the three categories of severity but not the two categories of clinical outcome. AKI diagnosed by KDIGO criteria but missed by RIFLE or AKIN was defined as [K(+)R(−)+K(+)A(−)]. Urine output data were not available in this study. Baseline SCr was estimated from either the admission value (if this was within the normal range) or if available [13]–[14], from another value within 3 months, whichever was lowest [15]–[17]. Patients with SCr elevation at the time of admission but without SCr record in the previous 3 months before admission were considered having AKI, according to the use of estimation of SCr by backward calculation from the Modification of Diet in Renal Disease (MDRD) equation as recommended [11]. In this study, baseline SCr within 3 months before admission accounted for 27%. SCr was measured by the same means of a kinetic Jaffé method in the 2 hospitals.

**Table 1:** Diagnosis and staging criteria for AKI of RIFLE, AKIN, KDIGO definitions based on SCr.

Classification	Definition for AKI	Stage	Serum Creatinine criteria for AKI staging^a^
RIFLE	Increase in SCr ≥50% within 7 d	Risk	To ≥1.5 times baseline
		Injury	To ≥2 times baseline
		Failure	To ≥3 times baseline or ≥44 µmol/L increase to at least 354 µmol/L
AKIN	Increase in SCr ≥26.5 µmol/L or ≥50% within 48 h	1	Increase of ≥26.5 µmol/L or to 1.5–2 times baseline
		2	To 2–3 times baseline
		3	To ≥3 times baseline or ≥26.5 µmol/L increase to at least 354 µmol/L or initiation of RRT
KDIGO	Increase in SCr ≥26.5 µmol/L within 48 h or ≥50% within 7 d	1	Increase in SCr ≥26.5 µmol/L within 48 h or to 1.5–2 times baseline
		2	To 2–3 times baseline
		3	To ≥3 times baseline or to at least 354 µmol/L or initiation of RRT

For patients meeting diagnosis criteria for AKI according to RIFLE, AKIN or KDIGO, the stage based on percentage increase were determined by the ratio of peak SCr value obtained during hospitalization to baseline. AKI: Acute Kidney Injury; RIFLE: Risk Injury Failure Loss ESRD; AKIN: Acute Kidney Injury Network; KDIGO: Kidney Disease Global Outcomes; SCr: serum creatinine; RRT: renal replacement therapy.

a Urine output was not used, because records of hourly urine output were not available in the majority of patients.

### Data Collection

Data including the patients' demographics, medical history (smoking, diabetes, hypertension, coronary heart disease and previous AHF), physical examination, etiology of AHF, medication use and biochemical parameters (including SCr on admission, or if available, SCr within 3 months prior to admission), length of stay (LOS) in the ICU and in the hospital, requirement for renal replacement therapy (RRT), and date of death were obtained from the hospitals' computerized database. On admission, baseline estimated glomerular filtration rate (eGFR) was calculated using MDRD equation [18]. Left ventricular ejection fraction (LVEF) was evaluated by Doppler echocardiography during hospitalization.

### Statistical analysis

Analyses were performed using the Statistical Package for Social Sciences software (SPSS, version 16.0). Continuous variables were expressed as means ± SDs or medians (with 25th and 75th percentiles) values, and were tested by the *t* test, Mann-Whitney U test or ANOVA, as appropriate. Categorical variables were described as numbers and percentages, and were compared using the chi-squared test. In-hospital mortality was estimated by the Kaplan-Meier method, and curves were compared with the log-rank test. Univariate and multivariate Cox proportional regression analyses were performed to assess the relationship between AKI and in-hospital mortality. Two-tailed P values <0.05 were considered significant.

## Results

### Study population characteristics

**Figure 1:**
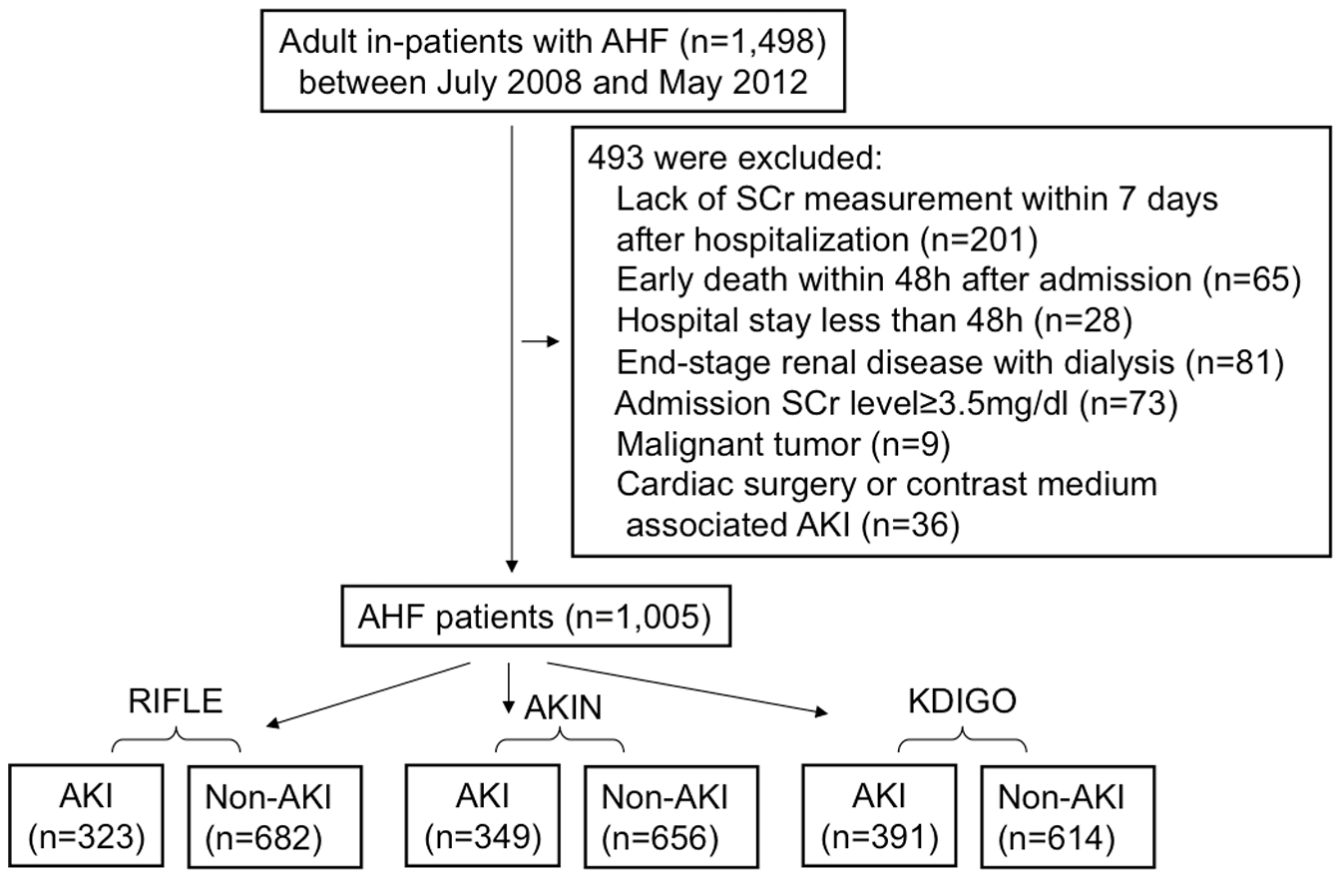
Schematic of study sample with exclusions from analysis.

Of the total of 1,498 patients, 493 patients were excluded from analysis (Fig. 1.). The final studied cohort was composed of 1005 subjects. The mean age was (68.5±15.0) years, and 439(43.7%) were women. The commonest etiology of AHF was ischemic heart disease (IHD) (43.8%), followed by valvular disease (22.6%) and cardiomyopathy (12.8%). 32.7% had diabetes and 27.5% had previous IHD. The mean eGFR was (59.1±24.9)ml/min/1.73 m^2^ at baseline, and mean LVEF, (49.3±12.9)%. The median LOS for hospitalization was 12(IQR: 8–20) days. 23.9% patients were admitted to ICU during hospitalization. A total of 64 patients (6.4%) were treated with RRT for AKI during hospitalization. Baseline characteristics stratified by AKI according to RIFLE, AKIN and KDIGO classification was described in Table 2. AKI group had more diabetic patients, lower eGFR, hemoglobin, serum albumin, higher C-reactive protein (CRP), less ACEI/ARB medication, more diuretic use and longer LOS in ICU.

**Table 2:** Demographic and clinical characteristics of patients with AHF according to the development of AKI by RIFLE, AKIN, KDIGO definitions during hospitalization.

Characteristic	RIFLE		AKIN		KDIGO	
	No AKI (n = 682)	AKI (n = 323)	No AKI (n = 656)	AKI (n = 349)	No AKI (n = 614)	AKI (n = 391)
Age(year)	68.6±15.0	68.3±15.1	68.4±15.2	68.8±14.8	68.4±15.3	68.8±14.7
Gender(Male) —n (%)	408(59.8)	158(48.9) *	381(58.1)	185(53.0)	360(58.6)	206(52.7)
Smoker—n (%)	173(25.4)	64(19.8)	164(25.0)	73(20.9)	154(25.1)	83(21.2)
Systolic BP (mmHg)	136±28	134±30	135±27	135±32	135±28	135±31
Diastolic BP (mmHg)	78±16	76±18	78±16	76±18	77±16	77±18
HR(beat/min)	90±21	93±21*	90±21	93±22	90±21	93±21
LVEF(%)	49.7±13.2	48.5±12.3	49.5±13.0	49.1±12.7	49.5±13.0	49.0±12.7
Hypertension—n (%)	446(65.4)	203(62.8)	422(64.3)	227(65.0)	395(64.3)	254(65.0)
Diabetes—n (%)	208(30.5)	121(37.5) *	194(29.6)	135(38.7) *	182(29.6)	147(38.4) *
Ischemic heart disease—n (%)	185(27.1)	91(28.2)	184(28.0)	92(26.4)	172(27.7)	104(27.2)
Causes of AHF						
Coronary artery disease	296(43.4)	144(44.6)	296(45.1)	144(41.3)	270(44.0)	170(43.5)
Valvular heart disease	161(23.6)	66(20.4)	147(22.4)	80(22.9)	142(23.1)	85(21.7)
Cardiomyopathy.	89(13.0)	40(12.4)	83(13.1)	46(13.2)	79(12.9)	50(12.8)
Hypertension	81(11.9)	43(13.3)	75(11.4)	49(14.0)	72(11.7)	52(13.3)
Congenital heart disease	37(5.4)	20(6.2)	35(5.3)	22(6.3)	35(5.7)	22(5.6)
Others	18(2.6)	10(3.1)	18(2.7)	8(2.3)	16(2.6)	12(3.1)
Admission eGFR (ml/min/1.73 m^2^)	58.8±24.1	59.9±26.5	61.3±23.1	55.2±27.5*	60.9±23.3	56.4±27.0*
Hemoglobin(g/L)	121±25	112±26*	123±24	110±27*	122±24	112±26*
Serum albumin(g/L)	32.7±5.9	31.7±6.1*	32.8±5.8	31.5±6.1*	32.8±5.9	31.6±6.1*
C-reactive protein(mg/L)	16.0 (7.9–46.0)	25.5* (11.8–67.0)	15.5 (7.9–43.8)	27.2* (12.0–72.1)	15.5 (7.5–43.6)	25.8* (11.8–68.3)
LDL-cholesterol(mmol/L)	2.5±1.1	2.4±1.1	2.4±1.1	2.5±1.1	2.4±1.1	2.5±1.1
ACEI/ARB medication—n (%)	477(69.9)	200(61.9) *	469(71.5)	208(59.6) *	435(70.8)	242(61.9) *
Diuretic medication—n (%)	527(77.3)	281(87.0) *	510(77.7)	298(85.4) *	473(77.0)	335(85.7) *
RRT—n (%)	14(2.1)	50(15.5) *	11(1.7)	53(15.2) *	10(1.6)	54(13.8) *
LOS in hospital (days)	13(9–21)	14(8–25)	14(9–21)	14(8–24)	12(8–20)	12(7–21)
LOS in ICU (days)	7(3–11)	6(3–12) *	7(3–11)	6(3–12) *	7(3–11)	6(3–12)*
ICU stay—n (%)	106(15.5)	134(41.5) *	105(16.0)	135(41.8)*	88(14.3)	152(38.9)*

AHF: acute heart failure; AKI: acute kidney injury; BP: blood pressure; eGFR: estimated glomerular filtration rate; LVEF: left ventricular eject fraction; ACEI: angiotensin converting enzyme inhibitor; ARB: angiotensin receptor blocker; RRT: renal replacement therapy; LOS: length of stay; ICU: intensive care unit.

*:compared with NO-AKI group, P<0.05.

### Identification of early CRS type 1

**Figure 2:**
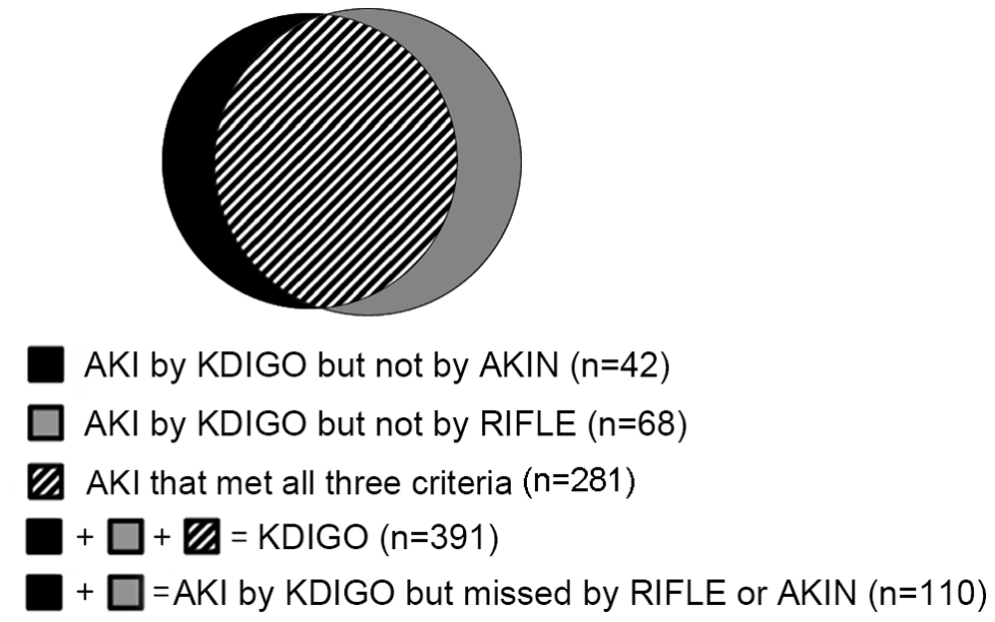
AKI distribution among RIFLE, AKIN and KDIGO classification.

Within 7 days, CRS type 1 that could be diagnosed by all three criteria occurred in 28.0% (281/1005). Incidence of CRS type 1 according to RIFLE, AKIN and KDIGO criteria was 32.1%, 34.7% and 38.9%, respectively. A total of 110 (10.9%) cases were diagnosed by KDIGO but missed by RIFLE or AKIN criteria (Table 3). KDIGO classification identified 68 more cases (17.4% in KDIGO) than RIFLE did and 42 more cases (10.7% in KDIGO) than AKIN did. 89.1% (98/110) of them were in either Stage 1 of AKIN or Stage Risk of RIFLE. The distribution of AKI patients among RIFLE, AKIN and KDIGO criteria was depicted in Fig. 2. and Table 4. Compared with patients in No-AKI group (by any criteria), the missed diagnosed AKI patients had lower hemoglobin, higher level of serum uric acid, low density lipoprotein (LDL), CRP and longer LOS in ICU. 87.2% patients developed CRS type 1 within two days of hospitalization.

**Table 3:** Incidence of CRS type 1 according to the RIFLE, AKIN, KDIGO and K(+)R(−)+K(+)A(−) definitions.

Classification	Incidence of AKI by all stages	Stage	Incidence of AKI by each stage
RIFLE	323(32.1%)	Risk	141(14.0%)
		Injury	116(11.5%)
		Failure	66(6.6%)
AKIN	349(34.7%)	1	157(15.6%)
		2	101(10.0%)
		3	91(9.1%)
KDIGO	391(38.9%)	1	194(19.3%)
		2	104(10.3%)
		3	93(9.3%)
K(+)R(−) + K(+)A(−)^a^	110(10.9%)	1	98(9.8%)
		2	3(0.3%)
		3	9(0.9%)

CRS: cardiorenal syndrome;

a : AKI diagnosed by KDIGO criteria but missed by RIFLE or AKIN criteria. AKI: Acute Kidney Injury.

**Table 4:** Concordance of AKI designation.

Definition	Comparison	No AKI by RIFLE or AKIN	AKI stage by RIFLE or AKIN		
			Risk/Stage 1	Injury/Stage 2	Failure/Stage 3
KDIGO	RIFLE				
NO AKI		614(61.1%)	0	0	0
Stage 1		61(6.1%)^a^	133(13.2%)	0	0
Stage 2		0^a^	0	104(10.3%)	0
Stage 3		7(0.7%)^a^	8(0.8%)	12(1.2%)	66(6.6%)
KDIGO	AKIN				
NO AKI		614(61.1%)	0	0	0
Stage 1		37(3.7%)^b^	157(15.6%)	0	0
Stage 2		3(0.3%)^b^	0	101(10.0%)	0
Stage 3		2(0.2%)^b^	0	0	91(9.1%)

AKI: acute kidney injury;

a : K(+)R(−), AKI diagnosed by KDIGO criteria but not by RIFLE criteria.

b : K(+)A(−), AKI diagnosed by KDIGO criteria but not by AKIN criteria.

### AKI and in-hospital mortality

The in-hospital mortality rate was 13.5% (136 patients). Hospital mortality in AKI patients was 23.5% by RIFLE, 23.8% by AKIN, and 23.5% by KDIGO criteria. A total of 18.4% deaths (25/136) occurred in the additional AKI group, of which 16 cases (11.8% of the overall death) were in K(+)R(−) group and 9 cases (6.6% of the overall death) in K(+)A(−) group. Those deaths in added AKI group by KDIGO criteria were mostly in Stage 1.AKI patients by four classifications, either by all stages or by each stage, showed higher mortality than patients without AKI (P<0.05). Hospital mortality stratified by four definitions showed increasing trends with stage progression. However, only AKIN and KDIGO criteria showed significance when compared Stage 1 to Stage 3 or Stage 2 to Stage 3 (Table 5).

**Table 5:** In-hospital mortality stratified by AKI or No-AKI.

Classification	AKI	No AKI
RIFLE(all stages)	76(23.5%)*	60(8.8%)
Risk	27(19.1%)*	-
Injury	28(24.1%)*	-
Failure	21(31.8%)* #	-
AKIN(all stages)	83(23.8%)*	53(8.1%)
Stage 1	28(17.8%)*	-
Stage 2	22(21.8%)*	-
Stage 3	33(36.3%)* ^#&^	-
KDIGO(all stages)	92(23.5%)*	44(7.2%)
Stage 1	36(18.6%)*	-
Stage 2	23(22.1%)*	-
Stage 3	33(35.5%)* ^#&^	-
K(+)R(−) + K(+)A(−)	25(22.7%)*	44(7.2%)
Stage 1	20(20.4%)*	-
Stage 2	1(33.3%)	-
Stage 3	4(44.4%)*	-

K(+)R(−)+K(+)A(−): AKI diagnosed by DIGO criteria but missed by RIFLE or AKIN.

*: compared with No-AKI group, *P*<0.05;

# : compared with Stage 1 or Stage Risk, *P*<0.05;

& : compared with Stage 2, *P*<0.05.

### Prognostic value of different AKI definitions on in-hospital mortality

**Figure 3:**
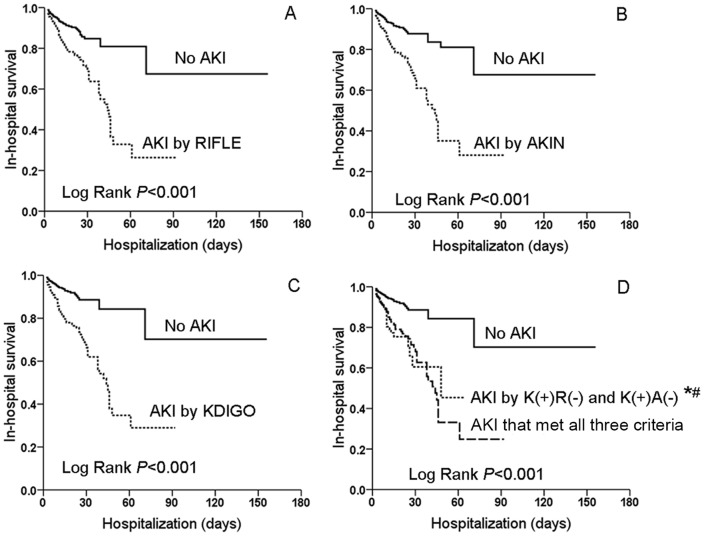
In-hospital survival of CRS type 1 according to RIFLE(A), AKIN(B), KDIGO(C) and K(+)R(−)+K(+)A(−) definitions(D). *: vs No-AKI, P<0.001;#:vs AKI by all three criteria, P = 0.061.

Kaplan-Meier curve showed AKI patients by RIFLE, AKIN, KDIGO and [K(+)R(−)+K(+)A(−)] definitions had lower hospital survival than non-AKI patients (Fig. 3., Log Rank P<0.001). After adjustment, the development of AKI by any definitions remained independently associated with in-hospital mortality. Odds ratio (OR) for RIFLE, AKIN, KDIGO and [K(+)R(−)+K(+)A(−)] was 2.56, 2,68, 4.00 and 3.24, respectively (*P*<0.05). By multivariable analysis, hospital mortality was also associated with stage progression by RIFLE, AKIN and KDIGO criteria (Table 6).

**Table 6:** Cox proportional analysis for in-hospital mortality in AHF patients.

	Unadjusted OR (95%CI)	*P* Value	Adjusted OR^c^ (95%CI)	*P* Value
RIFLE by all stages^a^	2.66(1.89–3.73)	<0.001	2.56 (1.79–3.65)	<0.001
RIFLE by each stage				
No AKI	reference		reference	
Risk	2.21(1.40–3.48)	0.001	2.24(1.41–3.56)	0.001
Injury	2.80(1.78–4.38)	<0.001	2.70(1.69–4.31)	<0.001
Failure	3.31(2.01–5.44)	<0.001	2.98(1.75–5.06)	<0.001
AKIN by all stages^a^	3.01(2.13–4.26)	<0.001	2.68(1.86–3.84)	<0.001
AKIN by each stage				
No AKI	reference		reference	
1	2.39(1.51–3.78)	<0.001	2.09(1.31–3.33)	0.002
2	2.85(1.73–4.69)	<0.001	2.72(1.62–4.55)	<0.001
3	4.07(2.63–6.30)	<0.001	3.69(2.31–5.89)	<0.001
KDIGO by all stages^a^	3.38(2.36–4.84)	<0.001	3.22 (2.24–4.62)	<0.001
KDIGO by each stage				
No AKI	reference		reference	
1	2.80(1.80–4.35)	<0.001	2.68(1.72–4.16)	<0.001
2	3.26(1.96–5.40)	<0.001	3.05(1.82–5.09)	<0.001
3	4.52(2.87–7.12)	<0.001	4.33(2.75–6.82)	<0.001
K(+)R(−) and K(+)A(−)^b^	3.47 (2.12–5.68)	<0.001	3.24 (1.97–5.35)	<0.001

AHF: acute heart failure; K(+)R(−)/K(+)A(−): AKI diagnosed by KDIGO criteria but missed by RIFLE and AKIN; OR: odds ratio; AKI: acute kidney injury;

a : including 1005 AHF patients;

b : including 724 AHF patients (614 without AKI and 110 with AKI diagnosed by KDIGO criteria but missed by RIFLE and AKIN criteria);

c : adjusted by gender, age, hemoglobin, serum albumin, medication with ACEI or ARB and diuretic and AKI.

## Discussion

The reported incidence of AKI after AHF (usually referred to as CRS type 1) varies widely depending on the definition used as well as the etiologies. Several studies have examined AKI in the context of AHF before the first consensus RIFLE criteria was proposed. Using the term ‘worsening renal failure (WRF)’ to describe the acute and/or subacute changes in kidney function that occurs following AHF, incidence of WRF ranged from 23–45% in acute decompensated heart failure (ADHF), and 9–19% in acute coronary syndrome [2], [19]–[23]. This broad incidence is mainly attributed to disparities in the definition used for WRF, difference in the observed time at risk and heterogeneity of selected populations [24]. Besides, there is no consensus on how best to define WRF, with some studies utilizing the absolute and others using relative changes in SCr values.

Data investigating the incidence of CRS type 1 was scarce after recommendation of RIFLE criteria. Most epidemiologic AKI data were from the subset of critically ill patients, sepsis patients or patients after cardiac surgery [25]–[27]. Only three studies evaluated the incidence of early CRS type 1.One retrospective study reported the incidence of AKI was 33% (125/376) in ADHF patients upon admission [28], and the other was 68.8% (344/500) in AHF patients admitted to ICU [29]. Both of these studies evaluated AKI by RIFLE criteria. In the very recent prospective study including 637 AHF admissions, AKI by four definitions (RIFLE, AKIN, KDIGO and WRF) was assessed and compared. AKI occurred in 25.6% by RIFLE, 27.9% by AKIN, 36.7% patients by KDIGO, and 33.0% by WRF [30]. In the present study, 28.0% patients met all three AKI criteria, and incidence of AKI was 32.1%, 34.7% and 38.9% by RIFLE, AKIN and KDIGO criteria, respectively. Our results showed the consistent epidemiology of early CRS type 1 with previous data in which AHF patients were in general ward but not in ICU [28]–[30]. Furthermore, 10.9% patients in our study were classified as AKI in KDIGO but not in RIFLE or AKIN criteria. Most of them were predominantly staged in the lowest AKI severity (Stage 1). This can be explained by the principles of AKI in KDIGO criteria.

In KDIGO criteria, only an absolute SCr increase of 0.3 mg/dl within 48 hs is sufficient for an AKI diagnosis, whereas in RIFLE, a SCr increase of ≥50% from baseline is necessary [11]. Compared with AKIN, KDIGO have an extended time frame to diagnose AKI. The combined use of small absolute increases in SCr and enough observation time in the KDIGO criteria may potentially make it more sensitive than RIFLE or AKIN, which therefore undoubtedly identified additional early CRS type 1 cases. However, those added cases may be less severe with lower mortality risk. Identification of more AKI cases may on one hand reduce misclassification, but on the other hand, increase risk dilution.

An important strength of our study was that we separated the additional diagnosed AKI by KDIGO to determine if the expanded proportion could predict short-term outcome of AHF. The mortality of missed diagnosed AKI by RIFLE or AKIN accounted for 18.4% in the total death and had a significantly longer stay in ICU, indicating a large number of patients with CRS type 1 and elevated risk for mortality would be missed by the RIFLE or AKIN criteria.

Interestingly, although the mortality of patients with AKI classified by RIFLE, AKIN and KDIGO criteria in our study was similar, the 3 classifications classified individual patients differently. The percentage of patients who were identified as non-AKI by the AKIN classification but fulfilled the RIFLE criteria for AKI was 4.2% (42/1005), among which 21.4% died (9/42). By contrast, 6.8% (68/1005) of patients were classified as non-AKI according to the RIFLE criteria but fulfilled the AKIN criteria for AKI, among which 23.5% died (16/68). From this standpoint, AKIN criteria missed less risk patients than RIFLE. KDIGO criteria detected all those high risk patients additionally. Mortality of the combined AKI group was nearly three times that of patients who did not have AKI by any criteria (22.7% vs 7.2%). Even after adjustment for covariables, the additional diagnosed AKI remained an independent risk factor for in-hospital mortality. These results suggest that KDIGO is a useful tool in clinical practice to identify “true AKI” because those diagnosed by this classification but missed by RIFLE or AKIN increased in-hospital mortality. Our results were consistent with Rodrigues's prospective study enrolling 1050 patients with AMI [31]. They compared incidence and mortality of AKI after AMI between KDIGO and RIFLE criteria and found that KDIGO criteria detected substantially more AKI patients than RIFLE. Patients diagnosed as AKI by KDIGO but not RIFLE criteria had a significantly higher early and late mortality. The author suggested KDIGO criteria were more suitable for AKI diagnosis in AMI patients than RIFLE. Using 3 criteria to compare their prognostic power, studies on cardiac surgery-related to AKI got the same results [32]. However, Roy et al's results demonstrate that the RIFLE and KDIGO classification systems have only marginally superior prognostic ability when compared to WRF and AKIN to predict the composite outcomes (death, re-hospitalization and RRT) [29]. One potential explanations for this discrepancy maybe they present no analysis of those additional diagnosed cased by KDIGO criteria. Altogether, there is little evidence assessing and comparing the three criteria so far.

Similar to other studies, our study found those missed diagnosed AKI patients who died during hospitalization were mostly in Stage 1, demonstrating even slight changes in SCr would have impact on prognosis in CRS type 1 patients. Small changes in SCr have also been associated with early and long-term mortality in cardiothoracic surgery patients, cohorts of AMI patients as well as other hospitalized individuals [33]–[35].

Although our findings confirmed the helpful utilization of KDIGO criteria, there still remain specificity limitations [36]. An important one is how to determine the baseline kidney function in which this baseline is not known. Whereas RIFLE and KDIGO suggest the use of back calculation, AKIN recommends using the evolution of SCr relative to the first observed value in that episode. The lack of a uniform approach to estimate this baseline has been recently shown to compound risk for AKI misclassification, hindering effective comparisons of this disease between settings [37]–[39]. In our study, patients with elevated SCr on admission but without previous SCr data were considered AKI according to a presumed ‘standard GFR’ of 75 ml/min/1.73 m^2^ as the KDIGO guideline recommended [11]. Another explanation was that SCr often increased at severe AHF presentation because of the hypoinfusion of kidney. However, those patients may have chronic kidney disease or acute-on-chronic kidney injury, which may overestimate the incidence of AKI.

There are several potential limitations in our study. Firstly, only the SCr criteria of AKI classification was evaluated, because urine output was difficult to collect in the general wards, and on the other hand, urine output was influenced by the diuretic therapy administered to the majority of AHF patients. Secondly, this was a retrospective study and only short-term prognosis was analyzed. Although a retrospective study had revealed relationship between the long-term prognosis of AHF and AKI [35], a prospective study with long-term follow-up will better demonstrate the implication by different AKI criteria. However, our study was so far the largest cohort to investigate the epidemiology and prognosis of CRS type 1 by KDIGO criteria.

In conclusion, the present study provided further insight into the epidemiology of early CRS type 1 using the new criteria KDIGO and revealed the association between AKI diagnosed by KDIGO but not RIFLE or AKIN and short-come prognosis. KDIGO criteria identified more episode CRS type 1 and predicted hospital survival. The clinical application of the KDIGO AKI definition helped to increase the early recognition of AKI, allowing more individuals at high risk of AKI for intervention, indicating the new KDIGO criteria were superior to RIFLE and AKIN criteria in predicting short-term outcomes in CRS type 1.

## Supporting Information

S1 Materials **Information of enrolled 1,005 AHF patients.**
(XLS)
